# Evaluation of Positioning Accuracy Using Smartphone RGB and LiDAR Sensors with the viDoc RTK Rover

**DOI:** 10.3390/s25133867

**Published:** 2025-06-21

**Authors:** Sara Zollini, Laura Marconi

**Affiliations:** 1 Department of Civil, Construction-Architectural & Environmental Engineering, University of L’Aquila, Piazzale E. Pontieri, 1, Monteluco di Roio, 67100 L’Aquila, Italy; sara.zollini@univaq.it; 2 Department of Engineering, University of Perugia, V. G. Duranti, 93, 06125 Perugia, Italy

**Keywords:** viDoc RTK Rover, smartphones, 3D survey, infrastructure monitoring, photogrammetry, LiDAR, GNSS positioning

## Abstract

Modern surveying is increasingly focused on fast data acquisition and processing using lightweight, low-cost equipment, particularly for the continuous monitoring of structures and infrastructures. This study investigates the use of LiDAR and RGB sensors embedded in Apple and Android smartphones, paired with an innovative device, the viDoc RTK Rover, for centimeter-level surveying. Three case studies were selected, each characterized by different materials, functional uses, and environmental contexts. The methodology centers on evaluating final accuracy during both the data acquisition and processing phases. Coordinates of target points were obtained directly via the viDoc device and indirectly through dense point clouds. Validation was conducted using a geodetic GNSS receiver. Results demonstrate that, in most cases, the system achieves accuracy comparable to traditional surveying methods. The findings confirm that these emerging tools offer a reliable and efficient solution for rapid 3D surveys with centimeter-level accuracy.

## 1. Introduction

Recent trends in geomatics research are increasingly focused on lightweight, affordable tools, particularly when constant monitoring of an object or phenomenon is required. Despite this growing interest, the academic literature has only just begun to explore the role of smartphones in surveying. Nonetheless, innovations over the past few years have demonstrated the considerable potential of smartphones not just as mobile data collectors, but as platforms capable of integrating advanced surveying technologies, using, for example, RGB (red, green, blue) and LiDAR (light detection and ranging) sensors in the photogrammetric process [1,2,3].

These technological advancements have made it possible to conduct high-accuracy surveys in a more accessible and flexible manner, with significant implications across multiple disciplines. Currently, most studies have focused on Apple devices, which have been employed in a variety of contexts, such as 3D reconstruction in mountainous regions [4], monitoring shoreline evolution [5], documenting cultural heritage [6,7], road mapping [8], agricultural assessments [9], and forest inventory [10,11].

One study [7] tested the performance of the iPad Pro and iPhone 12 Pro (Apple Inc., Cupertino, CA, USA), both equipped with LiDAR sensors, for cultural heritage applications. The authors evaluated three free iOS apps (SiteScape version 1.0.12, EveryPoint version 2.9, and 3D Scanner version 1.9.5) across three different scenarios. The findings suggested that the quality of 3D point clouds depended more on the software used than on environmental variables like lighting or surface materials. However, some limitations still require further investigation. Despite this, early research shows promising results. There is growing consensus that smartphones, particularly when paired with accessories like the viDoc RTK (real-time kinematic) Rover, can deliver accuracies comparable to traditional surveying systems [12,13,14]. The continuous evolution of satellite-based positioning systems has made GNSS (Global Navigation Satellite System) a core component in a wide range of applications [15]. Advances in equipment, services (like RTK networks), and algorithms have allowed low-cost GNSS receivers to spread widely, making precise positioning more accessible. Some papers even advocate that it would not always be necessary to use an external low-cost GNSS receiver to obtain precise RTK positions with smartphones [16]. Using these affordable devices in differential mode with CORS (continuously operating reference station) networks through network RTK (NRTK) services has become a reliable method, enabling high accuracy even with low-cost tools [17,18,19,20,21,22]. A recent study also assessed the use of an iPhone 14 Pro paired with the viDoc RTK Rover for road mapping purposes [8]. The research demonstrated that the generated LiDAR plus RGB point clouds achieved centimeter-level accuracy, indicating the system’s feasibility for extracting road-related data.

In summary, the advantages of these smartphone-based methods include the integration of both LiDAR and RGB sensors in a single device, better performance in low-light environments, minimal sensitivity to surface materials (unlike single-sensor setups, which struggle with glass or dark surfaces), and suitability for mapping small features. Moreover, the system is lightweight, cost-effective, and enables rapid data collection. On the downside, limitations include higher point cloud noise, lack of full control over the device parameters (due to limited technical documentation), and positional drift in large areas caused by inaccuracies in gyroscopic and accelerometer data, leading to reduced absolute accuracy (3–5 m).

To address some of the existing limitations, particularly those related to positioning accuracy, this study focuses on evaluating the performance of a recently developed device, the viDoc RTK Rover, when used in combination with smartphones. Although the viDoc is primarily designed for Apple devices, both Android and iOS smartphones were included in the tests for research purposes. The main objective was to assess the positioning accuracy achievable with this integrated system and compare it to a traditional geodetic GNSS receiver, which served as the reference. A series of target points was strategically positioned and detected using both the smartphone–viDoc system and the traditional GNSS receiver. In addition, outputs from photogrammetric processing, such as dense point clouds, were analyzed for further comparison. The distribution of these control points was planned to ensure homogeneous coverage across the test sites, with their quantity adjusted according to the area’s size and GNSS signal quality (i.e., larger areas or those with weaker signals required more points). The novelty of this research lies in demonstrating that lightweight and cost-effective tools can deliver centimeter-level accuracy, an advancement with potential significance for both researchers and practitioners, particularly those involved in the monitoring and preservation of vulnerable structures. In light of this, three different bridges were selected as case studies, each varying in material composition, intended use, and environmental context. At this initial stage, the primary objective was to evaluate positioning accuracy. While other environments with similar GNSS variability could have been selected, infrastructure-related sites were deliberately chosen as a foundation for developing future methodologies to support the maintenance and management of these vulnerable systems. By applying a consistent methodology across these varied scenarios, the study aims to ensure the replicability and broader applicability of its findings.

## 2. Materials and Methods

**Figure 1:**
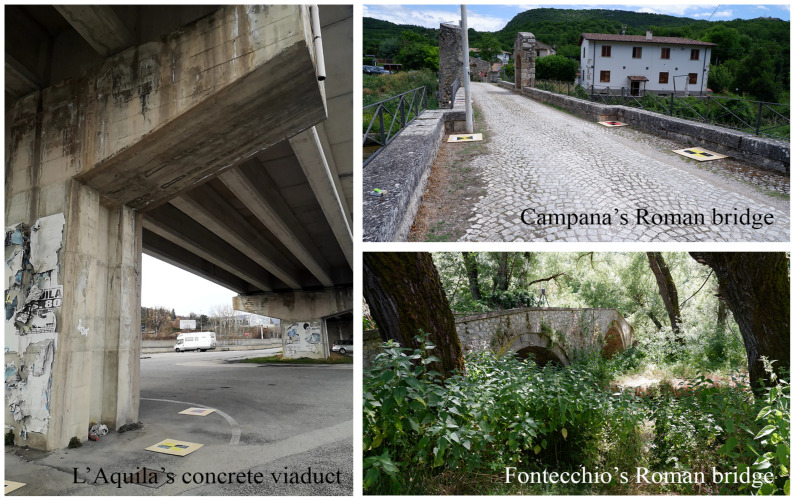
Case studies: concrete pillar of a viaduct in the city of L’Aquila, Roman bridge in the rural area of Campana, and the Roman bridge in the vegetated area of Fontecchio.

The case studies concern three bridges (Figure 1) that are composed of different materials, environments, and intended uses (diverse traffic loads).

The first viaduct analyzed in this study is situated in L’Aquila, the capital of the Abruzzo region in central Italy. Constructed between the late 1980s and early 1990s, the structure consists of nine spans made of pre-stressed concrete, extending over a total length of 225 m. Its supporting piers are monolithic elements built from reinforced concrete, with heights ranging from 4 to 6 m, depending on the surrounding topography. This viaduct plays a key infrastructural role, supporting heavy daily traffic as it links the western part of the city with its major commercial areas. The ability to access and walk beneath the structure made it possible to conduct a detailed survey in both open-sky and partially obstructed conditions in terms of GNSS signal performance. Beneath the central part of the surveyed area, satellite signal reception is limited due to the partial structural occlusion, while at the edges of the bridge, the sky visibility is notably better. For brevity, this case study will be referred to as the “L’Aquila’s viaduct” throughout the paper.

The second structure examined is a historic Roman bridge dating back to the 1st century AD. It serves as the sole access route to the small village of Campana, part of the municipality of Fagnano Alto, near L’Aquila. In ancient times, this bridge, along with several others in the region [23], formed a crucial connection between two major Roman settlements, Peltuinum and Alba Fucens, now key archaeological sites along the Apennines chain. Originally measuring approximately 10 m in length and 1 m in width, the bridge was constructed using a technique in which a fluid mortar was used to compress thousands of pebbles into a solid, waterproof, and highly durable mass. In the mid-19th century, two additional arches, each 3.60 m long, were added to either side of the original structure. More recently, the upper portion of the bridge was renewed, and although it remains accessible to vehicular traffic, it primarily serves a small population. Nowadays, the width ranges between 3 and 4 m. Campana currently has fewer than 50 residents and thus does not experience heavy or continuous use, unlike the previously described viaduct. The bridge is located in an unobstructed, open-air environment, ensuring clear visibility between the GNSS antenna and the sky. For brevity, this case study will be referred to as “Campana’s Bridge” throughout the work.

The third study site is located in the village of Fontecchio, a small town near Campana. This case focuses on an old stone bridge that spans the Aterno River just outside the village. Known locally as the “Stone Bridge”, it features a double-arched structure with asymmetrical spans and a crowned profile, constructed from limestone blocks. According to local accounts, the bridge was recently reinforced with concrete additions beneath the arches and along the upper curbs. Although it shares some structural similarities with Campana’s Bridge, it differs significantly in both setting and function. The bridge is largely surrounded and partially overgrown by dense vegetation, like willows and poplars, which poses potential challenges for both GNSS signal acquisition and photogrammetric 3D reconstruction. The vegetation does not form a completely opaque barrier, but satellite visibility remains challenging. Importantly, this is a disused structure, with no vehicular or pedestrian traffic currently using it. For consistency, this case study will be referred to as “Fontecchio’s Bridge” throughout the rest of the paper.

Collectively, the three bridges examined in this work vary in construction materials, environmental conditions, and functional roles. These distinctions allowed for the placement of control points and the application of different surveying technologies under a range of real-world conditions. The goal was to ensure that the analysis is not only comprehensive but also broadly replicable across diverse scenarios.

### Survey Materials and Methodology

**Figure 2:**
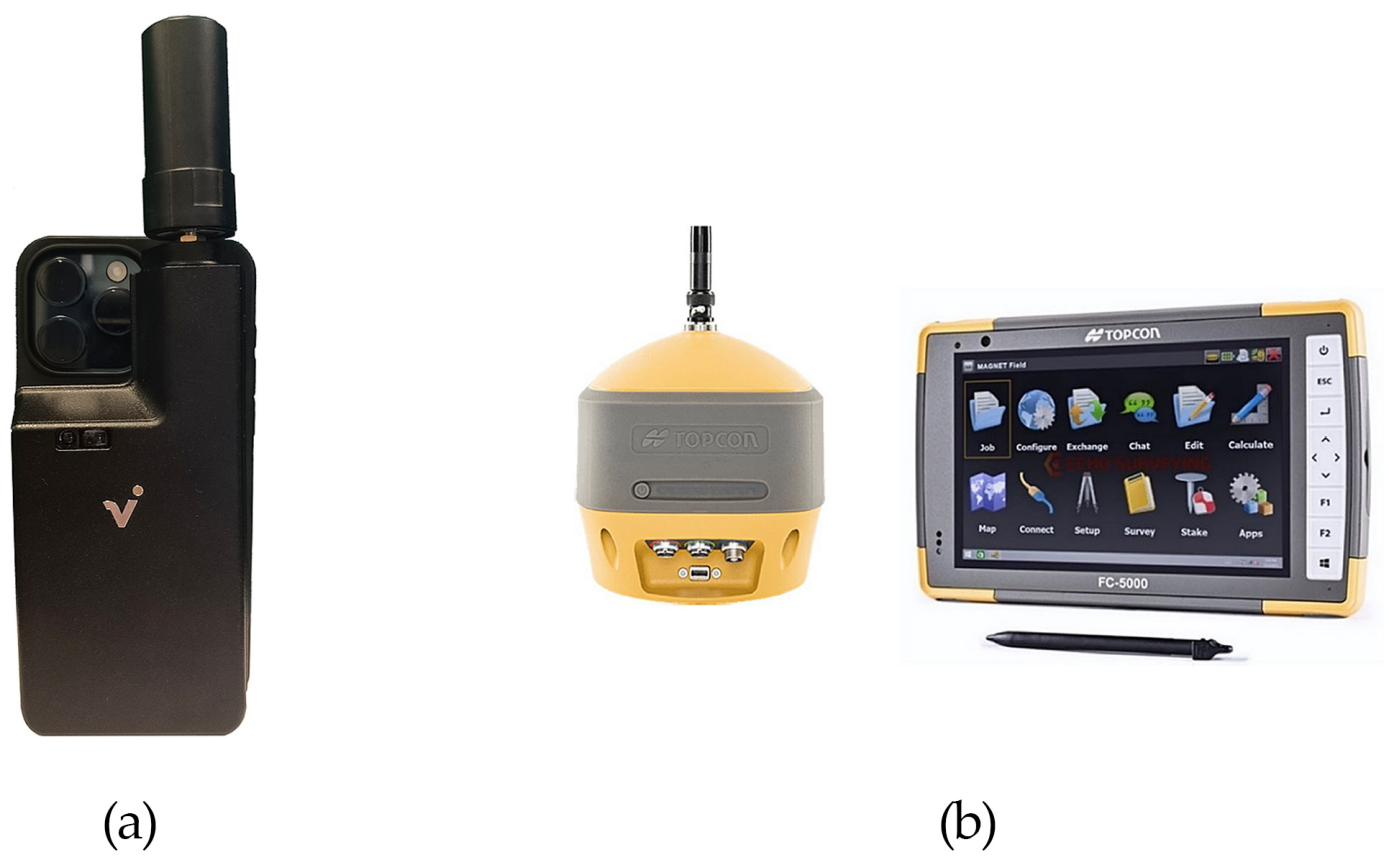
Representation of instrument settings: (**a**) Apple mobile phone with the viDoc RTK Rover and (**b**) high-precision geodetic GNSS receiver (HiPer HR by Topcon) (image credit: Topcon).

To acquire data, an Apple and an Android mobile phone were used (Table 1) equipped with the viDoc RTK Rover. The iPhone 14 Pro was selected for its native compatibility with the viDoc and its use of LiDAR and RGB sensors, enabling precise data capture. In contrast, the Xiaomi Redmi Note 12 Pro+ was used as a representative Android phone to test the viDoc RTK rover using only RGB data. The main goal was to assess the system’s versatility and positioning accuracy across devices with different hardware and operating systems. The target points were also acquired using a high-precision geodetic GNSS receiver (Figure 2).

The viDoc is a German-designed GNSS rover that connects to the Pix4Dcatch app for accurate site scanning on iPhone or iPad, with LiDAR used for photogrammetric data collection. The viDoc, which has an RTK antenna, fits on an Apple device with an SP Connect case. Once connected, the viDoc is paired with the PIX4Dcatch application and a user-designated NTRIP (Networked Transport of RTCM via Internet Protocol). PIX4Dcatch uses the mobile device’s camera to collect photogrammetric and LiDAR data, while the RTK antenna on the viDoc rover provides geotagged data with centimeter precision [24]. The classic geodetic GNSS receiver is a Topcon HiPer HR with an integrated antenna and equipped with a 9-axis inertial platform (IMU). Magnet Field software version 7.2 (by Topcon Positioning Systems) was used for performing an NRTK positioning, using HxGN SmartNet correction service.

**Figure 3:**
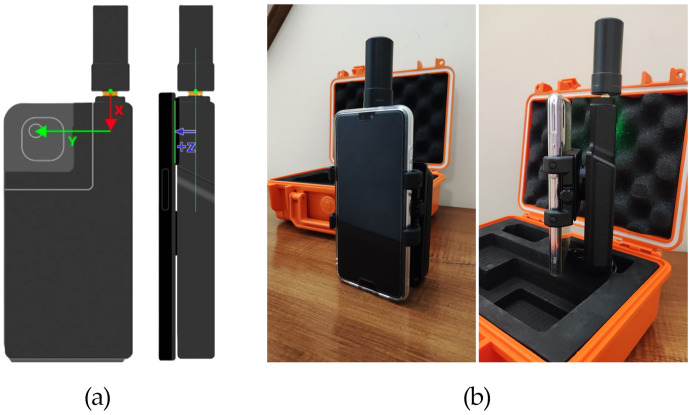
(**a**) X, Y, and Z axes to manually measure the camera offset (image credits [25]) and (**b**) configuration with Android mobile phone and universal SP Connect clamp with SPC+ connectors.

Since 2020, Apple has integrated both RGB and LiDAR sensors into its iPhone Pro and Pro Max series, whereas Android smartphones typically rely only on RGB cameras. As aforementioned, Apple does not publish official specs on the LiDAR sensor, but third-party developer documentation, research papers, and teardown analyses confirm that the LiDAR sensor integrated into the iPhone has a working range of about 0.2 m–5 m, with accuracy between 1 cm and 5 cm. ToF (time-of-flight) technology is used to measure distances with infrared waves, and its FOV (field of view) is between 70 and 80°. The viDoc RTK Rover connects to smartphones via Bluetooth; however, proper physical mounting is also required. For Apple devices, the viDoc is natively compatible, allowing for direct use with SP Connect cases. In this configuration, the system automatically detects and applies the camera offset between the GNSS antenna phase center and the camera’s optical center. In contrast, when using an Android device, such as a Xiaomi smartphone, an additional adapter is required. In this case, the camera offset must be manually measured and input along the X, Y, and Z axes to ensure accurate georeferencing of the camera’s optical center. Just to give an example, considering the first survey at L’Aquila viaduct, for Xiaomi, the manual offset was X=2.1 cm, Y=4.2 cm, and Z=3.5 cm. The adapter is a universal SP Connect clamp with SPC+ connectors (Figure 3).

**Figure 4:**
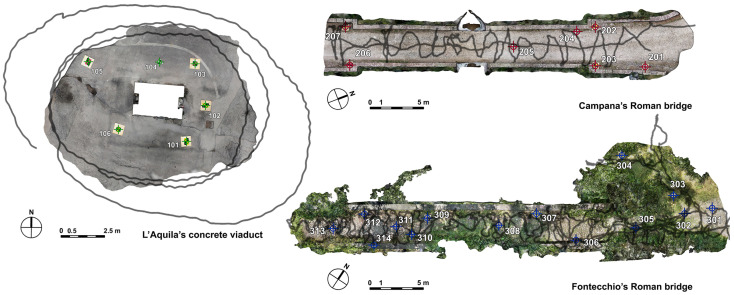
Representation of the walking path along the bridges and the target points, homogeneously distributed in the study areas, measured by traditional and new receivers.

PIX4Dcatch application, once connected via Bluetooth to the viDoc, shows the satellites used for RTK correction and asks for the NTRIP details, the mount point, and the coordinate reference system [26]. Images were captured following three loops around the pillar in L’Aquila, while for the other two cases, the surveys were conducted by walking along the bridges in a round-trip serpentine path (Figure 4).

**Figure 5:**
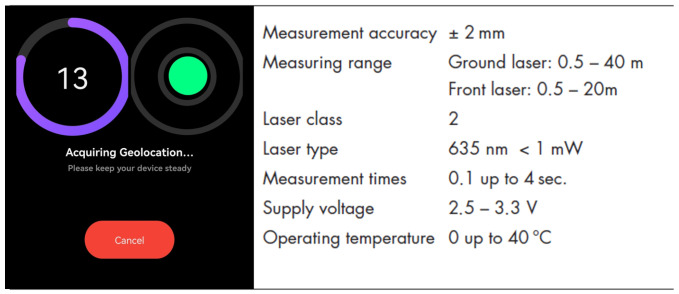
An example of point measurement with viDoc ground laser (GL) and relative technical specs [27].

Then, the geolocation information from the viDoc RTK Rover is tagged to the corresponding images based on the image timestamps. For this work, the NRTK corrections service was the HxGN SmartNet positioning services by Hexagon with VRS (Virtual Reference Station) corrections. Therefore, the baselines are short (a few meters). In the app, it is also possible to have an indication of the quality of data acquisition in the field, but it is more qualitative than quantitative. Therefore, as NMEA files are also created, they have been carefully analyzed for more scientific investigation. The viDoc also has a ground laser (GL) to measure the coordinates of specific points, and that was also tested in this work, together with the other receivers. It operates in conjunction with RTK GNSS positioning and an IMU. The laser sensor measures the distance from the device to the ground with an accuracy of ±2 mm over a typical range of 0.5 to 40 m. During point acquisition, the RTK GNSS provides precise coordinates, while the IMU determines the orientation of the device. Simultaneously, the laser sensor records the vertical distance to the target point on the ground. By integrating the GNSS-derived horizontal position, laser-measured distance, and orientation data from the IMU, the system calculates the three-dimensional (XYZ) coordinates of the measured point. In our case study, it was set to perform 10 acquisitions per second for a total of 15 s for each target point. A screen of how the point measurement happens and technical specs are reported in Figure 5. The stability is ensured by keeping the green circle inside the virtual bubble (Figure 5).

**Figure 6:**
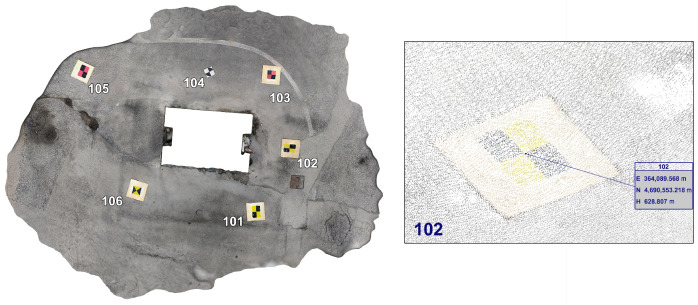
Example of point selection from a dense cloud.

All the equipment (mobile phone and viDoc), including one year of software licenses, has a value of approximately EUR 10,000, a value well below the orders of magnitude of traditional sensors, such as geodetic GNSS receivers, laser scanners, or UAVs with RGB or LiDAR sensors (mean value of around EUR 50,000 for hardware and software). If paid GNSS correction services are used, around EUR 500 per year should also be taken into account. For this reason, it is listed among the low-cost devices. The 3D coordinates have been measured not only from the direct survey using all the aforementioned receivers, but also from point cloud obtained by the photogrammetric process (widely used in literature for many purposes for decades [28,29,30,31,32,33,34,35,36,37]), in order to understand the reliability of the reconstruction (Figure 6).

An overlap among images of 90% was set during data acquisition. The reconstruction was performed using PIX4Dmatic software version 1.54.3. Three point clouds can be obtained: one from the RGB sensor, one from the LiDAR sensor (in the case of iPhone), and one from the fusion of the first two. After the alignment, the fusion is performed by a proximity-based merging, leveraging robust SfM (structure from motion) calibration and classic point-cloud matching. In order to fully test the capabilities of the viDoc positioning system, no GCPs were considered for the 3D reconstruction, to carry out rapid surveys and post-processing.

To sum up, the coordinates of target points (Figure 4), identified by colored markers homogeneously positioned around the survey areas, were measured considering the following:

- HiPer HR geodetic receiver (Topcon);
- viDoc ground laser (GL);
- Apple mobile phone with viDoc on dense point cloud;
- Android mobile phone with viDoc on dense point cloud.

All the coordinates were acquired in ETRF2000 and the outputs are displayed in ETRF2000/UTM Zone 33N. The constellations enabled during the data acquisition were GPS, SBAS, GALILEO, QZSS, GLONASS, BEIDOU, considering a cut-off angle of 10°. The survey with the viDoc lasted around 10 min for each mobile phone used in each case study.

## 3. Results

As is widely recognized, the classic geodetic GNSS receiver remains the most reliable instrument for determining point coordinates. For this reason, it is used as the ground truth in this study. One of the first key findings concerns the precision of the acquired data. In the current case studies, the geodetic GNSS receiver consistently achieved standard deviations of under 1 cm for the horizontal (planimetric) components and around 1 cm for the vertical (height) component at each measured point. Similarly, an analysis of the NMEA messages from the viDoc system across all three bridge sites showed that the coordinates were recorded with standard deviations of around 1 cm in both planimetry and height. This indicates that the precision of data acquisition with the viDoc is fully comparable to that of the traditional geodetic GNSS receiver. A more detailed assessment of positioning accuracy, particularly in relation to ground truth comparisons, is presented in Section 4.

In this section, the main results related to the three case studies (L’Aquila, Campana, and Fontecchio) are reported in Table 2. In this table, the percentage of solutions with fixed ambiguity, the number of images acquired by the sensors (iPhone RGB + LiDAR and Xiaomi RGB-only, both equipped with the viDoc RTK Rover for positioning enhancement), and the number of points in the dense (derived from RGB sensor) and the depth (derived from LiDAR sensor) clouds are summarized.

**Figure 7:**
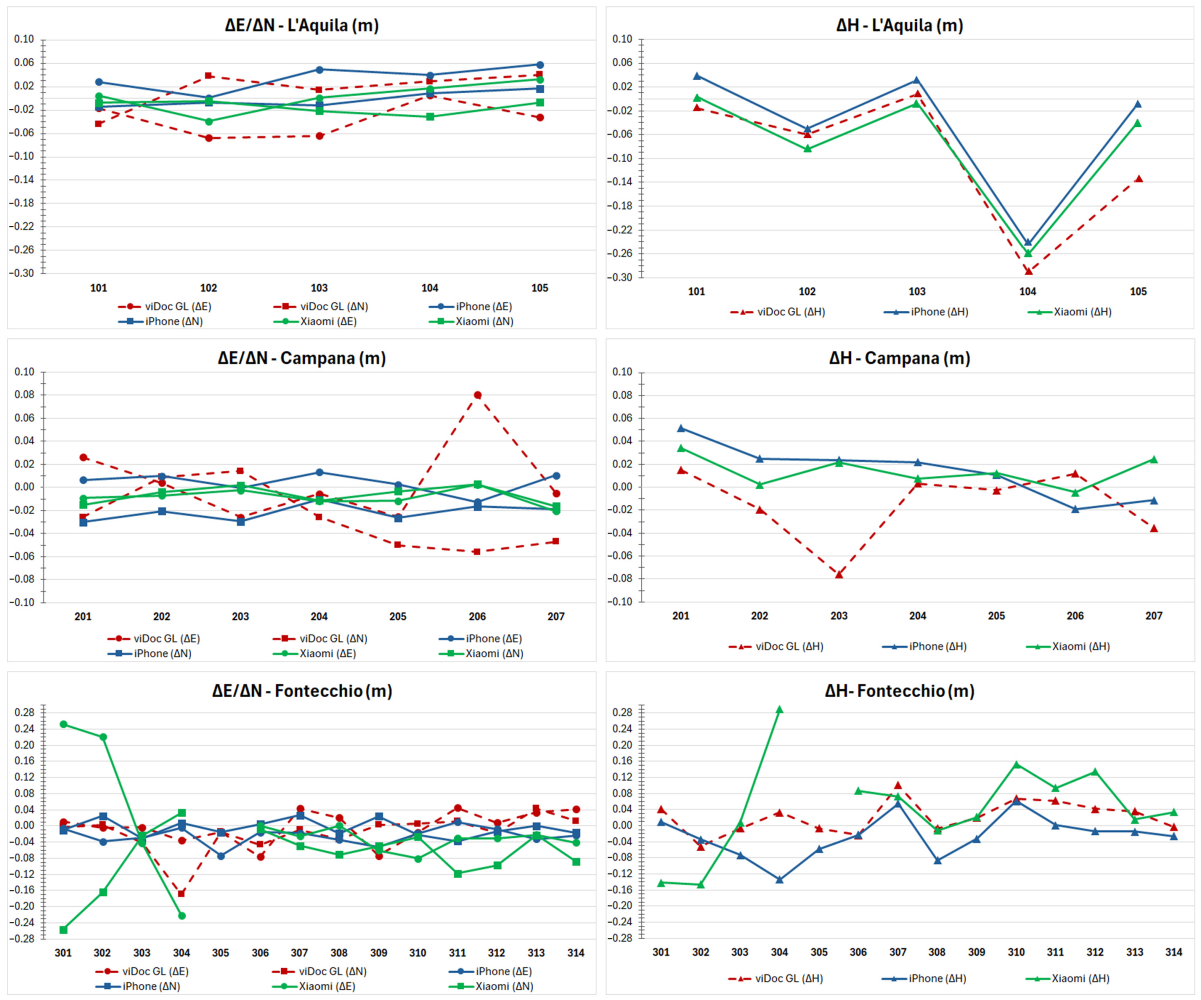
Graphical results related to the differences between the classic geodetic GNSS receiver and the other sensors for all case studies.

The differences in the east, north, and height components between the coordinates of the target points from the various sensors and the classic geodetic GNSS receiver (considered as ground truth), are calculated and reported both in graphical (Figure 7) and tabular formats (Table A1, Table A2 and Table A3 for L’Aquila, Table A4, Table A5 and Table A6 for Campana, Table A7, Table A8 and Table A9 for Fontecchio). The orthometric height was obtained by an interpolated geoid height (EGM08) of N=49.05 m, N=48.87 m, and N=48.86 m for the three case studies, respectively.

The graphs in Figure 7 were divided into planimetric and height components for each case study. On the *x*-axis, the number of target points is reported, while on the *y*-axis, the values of the calculated differences are reported. Red lines represent viDoc results; blue lines represent iPhone results; and green lines represent Xiaomi results. The dots refer to the east component, while the squares refer to the north component. The height is represented by triangles.

**Figure 8:**
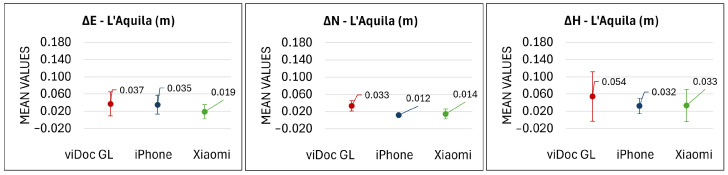
Means and relative standard deviations in the east, north, and height components in L’Aquila’s viaduct related to the coordinate differences between each sensor and the ground truth (HiPer HR). Along the *x*-axis, the names of the used sensors are reported; along the *y*-axis, there are the mean values.

For each case study, maximum, minimum, mean, and standard deviation values were calculated. Considering L’Aquila’s viaduct, the highest value was reached by viDoc GL with a value of 6.8 cm in the east and 4.4 cm in the north direction, while the minimum values are all under 1 cm, except the viDoc GL value in the north direction, which is, anyway, 1.5 cm. As is well known, the height deserves a separate discussion. Point 104 has a systematic error of around 25 cm, which will be discussed in Section 4. For this reason, it is not taken into account in this analysis. The maximum value is obtained by the viDoc GL (13.4 cm), and the minimum by the Xiaomi device equipped with the viDoc (0.4 cm). The mean values range between 1.2 cm and 3.7 cm in planimetry, and 3.2 cm to 5.4 cm in height (excluding point number 104), while standard deviations are on the order of around 1–2 cm in planimetry and between 2 cm and 6 cm in height (Figure 8).

**Figure 9:**
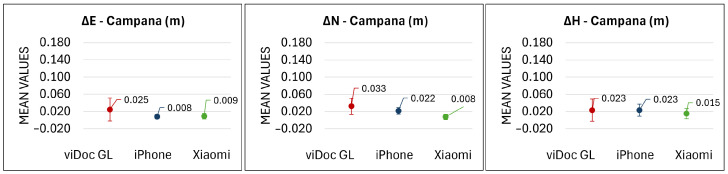
Means and relative standard deviations in the east, north, and height components at Campana’s Bridge, based on coordinate differences between each sensor and the ground truth (HiPer HR). The *x*-axis shows the names of the sensors used; the *y*-axis shows the corresponding mean values.

In Campana’s Bridge, the maximum value in the east direction is 8.1 cm, given by the viDoc GL, and the minimum value is 0.0 cm by an iPhone equipped with viDoc. In the north direction, maximum and minimum values are 5.6 cm and 0.2 cm, achieved by viDoc GL and Xiaomi plus viDoc, respectively. The mean values range between 0.8 cm and 3.3 cm in planimetry with standard deviations of around 1–2 cm (Figure 9). In height, the maximum is reached by the viDoc GL (7.6 cm) and the minimum is 0.3 cm, both by the viDoc GL and Xiaomi plus viDoc. The mean values with their standard deviations are both around 2 cm.

**Figure 10:**
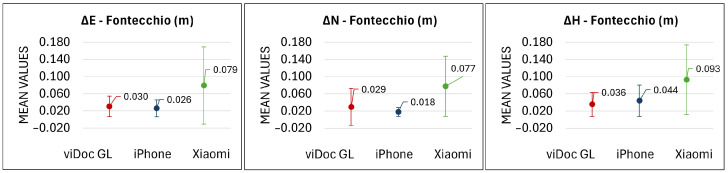
Mean values and relative standard deviations in the east, north, and height components at Fontecchio’s Bridge, based on the coordinate differences between each sensor and the ground truth (HiPer HR). The *x*-axis shows the names of the sensors used; the *y*-axis shows the corresponding mean values.

Considering the last bridge in Fontecchio, the maximum value in planimetry is reached by Xiaomi (25 cm in both east and north), while the minimum is for all tests under 1 cm. The mean values are around 2–3 cm for viDoc GL and iPhone plus viDoc, while Xiaomi shows values of around 8 cm. The standard deviations range between 1 cm and 9 cm. In height, the maximum value is 28.9 cm (Xiaomi) and the minimum is 0.1 cm (iPhone). The mean values range between 3.6 cm and 9.3 cm (standard deviations range between 2.8 cm and 8.1 cm) (Figure 10).

**Figure 11:**
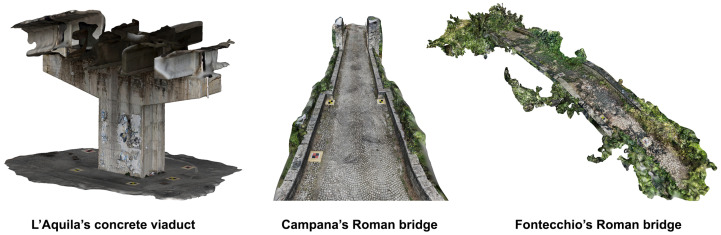
The 3D models of the viaduct in L’Aquila and the Roman bridges in Campana and Fontecchio acquired using mobile phones equipped with the viDoc RTK Rover and processed with PIX4Dmatic.

The final 3D models of the viaduct in L’Aquila and the Roman bridges in Campana and Fontecchio are shown in Figure 11.

## 4. Discussion

This section provides an in-depth discussion of both the limitations and strengths of using the viDoc RTK Rover in combination with Apple and Android smartphones. The first aspect analyzed concerns the final positioning accuracy, specifically the proportion of fixed RTK solutions obtained for each device across the three case studies. At the L’Aquila’s viaduct, both the iPhone and the Xiaomi smartphone encountered difficulties in maintaining fixed solutions beneath the structure, particularly on the western side, where a temporary loss of signal was likely. One potential explanation could be the inclination of the antenna during data acquisition. However, the viDoc RTK Rover is equipped with an omnidirectional antenna that should retain signal integrity at tilts up to 45° from the nadir. In all three test sites, special care was taken to keep the device as vertical as possible during measurement. To further investigate performance differences, a third device, a Huawei P20, was tested at this location. In this scenario, it achieved 94% fixed solutions, compared to 42% and 70% for the iPhone and Xiaomi, respectively. When comparing the Xiaomi and Huawei results, the lower performance of the Xiaomi device may be attributed to differences in mobile network services: the Xiaomi device operated via a mobile virtual network operator (MVNO), while the Huawei device used a mobile network operator (MNO). The quality of network-based corrections, critical for maintaining fixed RTK solutions, depends on the continuity and latency of data transfer. Huawei’s superior performance suggests a more stable and reliable data connection due to the MNO. However, the same explanation does not apply to the discrepancy between the iPhone and Huawei, as both used the same service provider. In this case, a temporary loss of signal in the urban environment of L’Aquila appears to be the most plausible cause. Notably, such issues did not occur in the other two case studies. As a potential mitigation strategy, future work could explore the effectiveness of different NRTK correction services in urban environments. In the rural setting of Campana’s Bridge, the open-sky conditions and lack of interference made it an ideal testing environment. All devices achieved nearly 100% fixed solutions, confirming the expected performance under optimal conditions. At Fontecchio’s bridge, despite the dense vegetation surrounding the site, the iPhone and Xiaomi performed well, achieving 93% and 80% fixed solutions, respectively. Signal disruptions occurred only at the lower portion of the bridge, where the vegetation was densest. However, this had no significant impact on the overall accuracy or quality of the results.

The observed differences in the number of images acquired by the smartphones can be attributed primarily to sensor characteristics, encompassing both hardware capabilities and software optimization, particularly during continuous scanning processes. Apple’s iPhone Pro series is equipped with advanced image signal processors (ISPs) integrated into its A-series chips. These ISPs are specifically designed for high-efficiency image processing, enabling rapid frame capture and smooth handling of large volumes of image data. From a hardware perspective, the iPhone sensors offer fast readout speeds, allowing image data to be quickly transferred to the processor. On the software side, iOS is known for its seamless integration between hardware and software. This synergy ensures that the sensors and processing units function optimally even during high-demand operations. Furthermore, Apple devices store images in the HEIC format, which provides high compression efficiency with minimal quality loss, facilitating faster and more memory-efficient storage. Overall, Apple devices benefit from a tightly integrated architecture where RAM, processor, and storage are highly optimized for performance, which typically results in faster image acquisition than many Android devices, which often suffer from fragmented hardware-software configurations. However, recent advancements in Android smartphones are narrowing this performance gap [38]. Another noteworthy observation during fieldwork was the iPhone’s superior thermal management during prolonged use. Better heat dissipation allowed it to maintain consistent performance throughout continuous scanning tasks. Nonetheless, acquiring a higher number of images does not necessarily mean an improvement in accuracy. In fact, an increased image count can also introduce more noise into the dataset. Thus, a careful balance must be struck between achieving a detailed 3D reconstruction and minimizing noise, an issue further explored in the next section. In addition, the speed of acquisition also plays a crucial role in terms of noise reduction. Being aware of the trade-off between overlap and noise, future work could explore optimizing the overlap percentage to balance image redundancy with spatial coverage and matching robustness.

**Figure 12:**
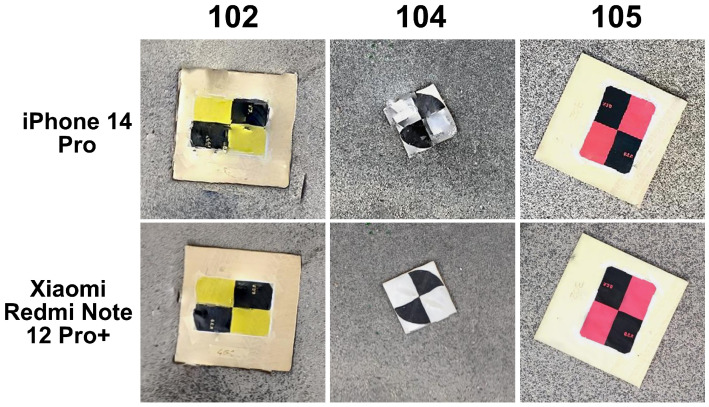
Example of three target points taken on the point clouds reconstructed from Apple and Android mobile phones. As can be seen, not all the target centers can be easily established.

Another relevant aspect worth discussing is the comparison of target identification on dense point clouds. In general, the selection of accurate target points is not straightforward; the main source of uncertainty comes from the inherent noise of the point clouds themselves. While a higher number of acquired images often results in greater detail and richer reconstruction, it simultaneously introduces increased noise. This can complicate the process of visually identifying and selecting precise targets. For instance, in the case of L’Aquila’s viaduct, the iPhone produced a highly detailed, dense cloud. However, this level of detail also introduced visual clutter, making it difficult to clearly discern the center of the selected targets. An example is illustrated in Figure 12. It becomes evident that not all target centers are easily identifiable due to the point cloud’s density and noise, highlighting the challenge of ensuring precision in visual-based measurements.

**Figure 13:**
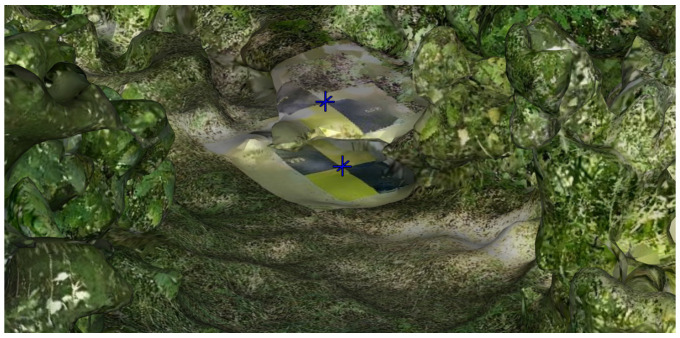
Example of the ambiguity created during the reconstruction of a few target points positioned in areas completely vegetated. The blue marker indicates the ambiguity of the target center.

For a more complete analysis, target coordinates were extracted from both the dense cloud and the 3D model. The differences between these datasets were evaluated using maximum, minimum, and mean values, along with their standard deviations. The results indicate that these differences generally fall within the precision limits of the data acquisition process (approximately 1 cm), as expected. The 3D model is a product of the point cloud, not an independent source, but it is worth noting that in challenging areas, like the Fontecchio site, the high density of vegetation, in addition to interpolation and texture rectification errors, posed challenges for mesh generation and texture mapping, resulting in increased uncertainty (Figure 13).

When comparing the mobile phones across the three regions of interest, the minimum and mean values obtained from the iPhone are comparable to those from the Xiaomi device, as are the corresponding standard deviations. In all cases, the observed differences remain below the measurement error associated with the employed technique. Furthermore, this analysis indicates that the number of acquired images does not have a substantial impact on the final precision.

**Figure 14:**
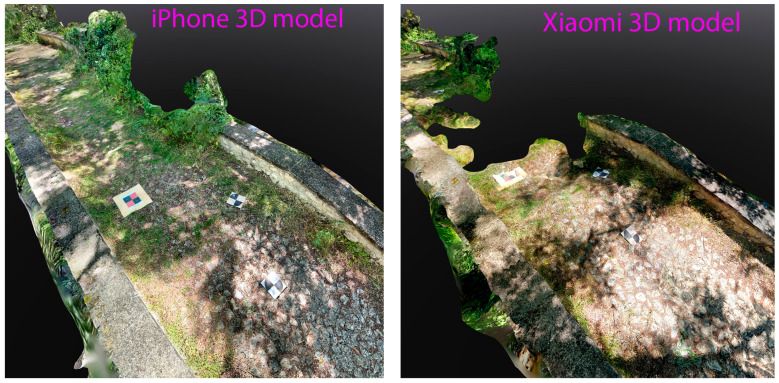
Portion of 3D models obtained by iPhone and Xiaomi in the vegetated area of Fontecchio. The more efficient iPhone-viDoc system helped the 3D reconstruction, where Xiaomi provides “holes” in both vegetated and non-vegetated parts.

An analysis of the 3D reconstructions reveals that the most challenging case study, as anticipated, was the Fontecchio bridge. In contrast, the rural setting of the Campana bridge represented an ideal condition, free from obstructive elements such as overhead structures (as in L’Aquila) or dense vegetation (as in Fontecchio). In Campana, the mobile device choice had minimal impact on the reconstruction quality. In L’Aquila, issues were observed primarily in the area beneath the bridge. The 3D model generated by the iPhone was likely affected by the higher point density, which introduced increased noise. A more balanced outcome, minimizing both image count and point cloud noise, was achieved using the Xiaomi device. Notably, even in the shaded areas beneath the bridge, the target centers were clearly identifiable in all reconstructions (Figure 12). Fontecchio required a different approach due to the presence of dense vegetation. In this case, the combination of dense and depth clouds helped the reconstruction. A higher number of images provided by the iPhone was also crucial to enable the reconstruction of both vegetated parts of the bridge and target markers, albeit with some limitations in areas of denser vegetation, where there can be ambiguity in the target point center selection (Figure 13). The Android phone (Xiaomi) resulted in visible gaps in the final model, particularly in vegetated zones (Figure 14).

For further comparison, the Huawei P20 (Huawei Technologies Co., Ltd., Shenzhen, Guangdong, China) was also tested. However, due to its lower RAM (4 GB) and older GPU (Mali-G72 MP12), it experienced significant overheating. This was likely caused by the high computational effort required to identify tie points between images in order to maintain the established 90% overlap. As a result, the device halted data acquisition and required multiple working sessions to achieve acceptable outcomes. In such cases, it is therefore advisable to use a LiDAR sensor combined with RGB sensors on a device equipped with at least 6 GB of RAM.

The core of this work is the comparison of coordinate differences between points coordinated with GNSS, used as ground truth, and the same points coordinated by means of the other tested sensors and products (see Table A1, Table A2, Table A3, Table A4, Table A5, Table A6, Table A7, Table A8 and Table A9). Initial data analysis was conducted to identify and exclude any gross errors. In the first case study in L’Aquila, an issue was encountered with point 106, where the geodetic GNSS receiver recorded coordinates significantly different from those obtained by all other sensors (nearly 1 m in planimetry and about 3 m in height). Examination of the raw data revealed that this point had a very high GDOP value (around 20, whereas a typical range is between 2 and 6) and was based on a limited number of visible satellites (approximately 10, about half the number used in other measurements). This was considered a gross error, and point 106 was excluded from further analysis to avoid distortion of results. A similar issue was noted with point 104, but only in the height component. Therefore, this point was retained in the analysis. As shown in Table A3, all sensor height differences for point 104 show a systematic error of approximately 25 cm, suggesting an error occurred during acquisition with the HiPer HR receiver. In the Campana and Fontecchio case studies, no such anomalies were observed. However, at the Fontecchio bridge site, vegetation caused some interference. No acquisition problems occurred with the iPhone, but the Xiaomi device failed to correctly acquire data for several target points (see Table A7, Table A8 and Table A9). For this reason, a higher number of points was surveyed in Fontecchio compared to the other two sites.

To assess the accuracy, the maximum, minimum, mean, and standard deviation of the coordinate differences were calculated for all three bridges. At the L’Aquila viaduct (Figure 8), the mean values achieved using viDoc paired with smartphones were comparable to those of traditional GNSS systems. The use of only the ground laser, however, resulted in slightly lower performance. These results demonstrate the viDoc’s effectiveness for surveying objects beneath viaducts, particularly in planimetric accuracy. Similar observations apply to Campana’s bridge. As shown in Figure 9, in open-air conditions, both Apple and Android systems achieved accuracy levels within or close to the expected measurement error when used with the viDoc. However, when relying only on the viDoc’s ground laser, the deviations were slightly greater than those from traditional systems. Finally, the results from the Fontecchio bridge are analyzed. The more complex and vegetated environment likely increased the difficulty of data acquisition. Despite lower performance compared to the other two sites, the results remain promising. Accuracy was generally consistent with reference values, except for the Xiaomi device, which exhibited a mean deviation of 7 cm, so slightly higher than traditional systems. The analysis revealed that, in vegetated areas, the point cloud derived from the Xiaomi device lacked many points, and those that were detected showed the highest errors. In contrast, when using the iPhone-viDoc system, no data were lost, and the achieved accuracy remained within the expected measurement error.

In conclusion, across all three case studies, the results obtained using lightweight and low-cost sensors were encouraging. Although deviations of up to 30 cm from the geodetic GNSS receiver were observed in some occluded areas, in open or partially occluded environments, all sensors achieved accuracies within a few centimeters. In vegetated environments, the use of the RGB-only sensor on the Xiaomi device resulted in significantly higher errors, while the iPhone, which has both LiDAR and RGB sensors and is optimized to work with the viDoc system, could achieve results closer to the reference values. Moreover, considering that the viDoc RTK Rover is designed primarily for open-air use, these findings suggest that it can also perform effectively in partially and fully occluded environments.

## 5. Conclusions

This paper focuses on the use of Apple and Android mobile systems equipped with an innovative device, the viDoc RTK Rover, to assess its reliability in comparison with traditional positioning systems, specifically a geodetic GNSS receiver used as ground truth. The Apple device (iPhone 14 Pro) uses both RGB and LiDAR sensors, while the Android device (Xiaomi Redmi Note 12 Pro+) relies on an RGB sensor. The viDoc is designed to enhance positioning accuracy to the centimeter level. Three case studies were carried out, each involving a different bridge that varies in construction materials, intended use, and surrounding environment. The primary objective of the study is to evaluate the reliability of these mobile systems in different environments, in combination with the viDoc, for extracting the 3D coordinates of specific target points.

The first environment is an area partially occluded by spans of a modern viaduct made of concrete, designed for heavy traffic, and located in an urban setting. The second bridge, an ancient Roman structure built of stone, is in a rural open-air scenario with no interference, and is occasionally used by vehicles. The third one, another Roman stone bridge, is situated in a heavily vegetated area, partially covered by shrubs and surrounded by tall trees, with no current vehicular load. Target points were homogeneously distributed across each study area. The 3D coordinates of these points were acquired using both the geodetic GNSS receiver (as reference) and the viDoc’s integrated ground laser. Additionally, coordinates were extracted from dense point clouds generated via photogrammetric processing. Results and discussion are organized into three main thematic areas:

1. Analysis of data acquisition, in terms of how many fixed solutions have been achieved from each mobile phone;
2. Comparison of the final 3D reconstruction in the three different environments;
3. Analysis of coordinate differences between the viDoc RTK Rover outputs with the geodetic GNSS receiver.

The results indicate that, across all three test areas, high percentages of fixed RTK solutions were achieved, ranging between 70% and 100%, with the exception of the iPhone in the first case study. This anomaly did not occur in the other two locations, suggesting that the likely cause was a temporary signal loss due to the urban setting of the viaduct in L’Aquila. In general, the Xiaomi device consistently delivered slightly lower accuracy compared to the iPhone. This discrepancy is likely attributed to the type of service provider used; Xiaomi operates through a Virtual Mobile Network Operator (VMNO), which may result in reduced continuity and integrity of the correction data received.

The quality of the final 3D reconstructions also varied by environment. In the rural and open-air site of Campana, the 3D model showed no significant issues. In the partially occluded urban viaduct in L’Aquila, a good balance between computational efficiency and noise level was achieved using the Xiaomi device. In contrast, at the Fontecchio site, Apple devices performed better. The dense vegetation presented a considerable challenge, particularly for Xiaomi. The Apple device’s use of both RGB and LiDAR sensors, combined with its greater processing power, superior stabilization, and likely better software optimization, enabled a complete and accurate reconstruction of the scene. In comparison, the Xiaomi device struggled to generate a reliable 3D reconstruction, resulting in numerous gaps, especially in vegetated areas and around targets partially obscured by foliage.

The comparison between the new viDoc RTK Rover and a traditional geodetic GNSS receiver yielded promising results. In terms of precision, both systems consistently achieved values around 1 cm during data acquisition. When analyzing accuracy, most target points showed deviations from the ground truth that fell within the expected measurement error. While the viDoc ground laser (GL) tended to show slightly higher differences, the values remained acceptable for practical surveying purposes. The Fontecchio site posed the greatest challenge due to dense vegetation. Here, using the Xiaomi device, the largest mean discrepancies observed were 7.9±9.0 cm in planimetry and 9.3±8.1 cm in height. However, when using the iPhone, the mean differences were significantly reduced to around 2.5±2.5 cm, closely aligning with reference values. Given the same trajectory, using two different sensors (RGB and LiDAR) helped the reconstruction, but the fundamental point is that Apple devices are optimized with the viDoc RTK Rover. Moreover, greater processing power and more effective stabilization also contribute to higher efficiency in the entire process.

This study underscores the transformative potential of mobile devices in the field of geomatics. While traditional systems still outperform in particularly complex or demanding scenarios, smartphones paired with positioning-enhancement tools like the viDoc RTK Rover represent a valuable, more accessible, and cost-effective alternative. Their lightweight and relatively low-cost nature makes them particularly suitable for rapid surveys, especially in areas with limited access to resources or in applications requiring frequent monitoring, such as infrastructure inspections. One of the most significant advantages is the use of RGB and LiDAR sensors in a compact and portable instrument. These widely available mobile devices can be seamlessly integrated into GNSS-based workflows, leveraging real-time corrections from GNSS networks to achieve high positional accuracy. This capability enables professionals to frequently inspect critical infrastructures, such as bridges and roads, by quickly generating accurate, georeferenced 3D models, thereby supporting timely and informed decision-making in asset management. The logistical simplicity and portability of this system can also be crucial in emergency scenarios, where real-time damage assessment is essential. In such cases, fast initial surveys can be conducted immediately after the event, with centimeter accuracy.

Two main future studies are planned. The first is a comparative analysis of point clouds generated by smartphones (equipped with viDoc) and those produced by traditional systems such as UAVs, terrestrial photogrammetry, and laser scanning. This will help further quantify the accuracy and usability of mobile solutions in professional surveying contexts. The second one is automated decay detection using AI. The development of a reliable methodology to detect and classify decay forms in infrastructure, such as cracks, erosion, or spalling, using point clouds or 3D models generated by smartphones and the viDoc system, is crucial for infrastructure management and safety. This study aims to integrate deep learning and artificial intelligence techniques to support preventive maintenance and improved management of transportation networks and critical infrastructure.

## Figures and Tables

**Table 1:** Smartphone (iPhone 14 Pro, Apple Inc., CA, USA, and Xiaomi Redmi Note 12 Pro+, Xiaomi Inc., Pechino, China) specifications used with the viDoc RTK Rover for this work.

Model	System	RAM	GPU	Sensor	Focal Length	Sensor Size	Pixel Size
iPhone 14 Pro	iOS	6 GB	Apple GPU (5-core)	RGB + LiDAR	3.7 mm	RGB 1/1.28″ LiDAR 3.5 mm	RGB 1.22 μm LiDAR N/A
Xiaomi Redmi Note 12 Pro+	Android	8 GB	Mali-G68 MC4	RGB	3.4 mm	1/1.4″	0.56 μm

**Table 2:** Results related to the three case studies (L’Aquila, Campana, and Fontecchio): percentage of solutions with fixed ambiguity; number of images acquired by the sensors (iPhone RGB + LiDAR and Xiaomi RGB-only); and number of points in the dense point cloud (derived from the RGB sensor) as well as the depth point cloud (derived from the LiDAR sensor).

		% of Fixed Solutions	n° Images	n° Points Dense Cloud	n° Points Depth Cloud
L’Aquila	iPhone	42	1185	38,010,187	2,170,942
	Xiaomi	70	315	2,860,769	-
Campana	iPhone	100	1467	76,275,660	3,696,325
	Xiaomi	100	1044	6,975,164	-
Fontecchio	iPhone	93	3464	99,451,405	8,616,626
	Xiaomi	80	2153	8,232,396	-

## Data Availability

Data are available upon request to the authors.

## Abbreviations

The following abbreviations are used in this manuscript:

3D	Three-Dimensional
AD	Anno Domini
CORS	Continuous Operating Reference Stations
EGM	Earth Gravitational Model
ETRF	European Terrestrial Reference Frame
FOV	Field of View
GCP	Ground Control Point
GDOP	Geometric Dilution Of Precision
GL	Ground Laser
GLONASS	GLObalnaya NAvigatsionnaya Sputnikovaya Sistema
GNSS	Global Navigation Satellite System
GPS	Global Positioning System
GPU	Graphics Processing Unit
HEIC	High-Efficiency Image Container
HR	High Resolution
IMU	Inertial Measurement Unit
ISP	Internet Service Provider
LiDAR	Light Detection And Ranging
MNO	Mobile Network Operators
MVNO	Mobile Virtual Network Operators
NMEA	National Marine Electronics Association
NRTK	Network Real-Time Kinematic
NTRIP	Networked Transport of RTCM via Internet Protocol
QZSS	Quasi-Zenith Satellite System
RAM	Random Access Memory
RGB	Red Green Blue
RTCM	Radio Technical Commission for Maritime Services
RTK	Real-Time Kinematic
SBAS	Satellite-Based Augmentation System
SfM	Structure from Motion
ToF	Time-of-Flight
UAV	Unmanned Aerial Vehicle
UTM	Universal Transverse Mercator
VRS	Virtual Reference Station

## Appendix A

This section presents the results related to the differences between the coordinates obtained from the ground truth (provided by the geodetic GNSS receiver) and all viDoc-related measurements in L’Aquila (Table A1, Table A2 and Table A3), Campana (Table A4, Table A5 and Table A6), and Fontecchio (Table A7, Table A8 and Table A9).

**Table A1:** Differences between the coordinates acquired by the viDoc ground laser (GL) and mobile phones with viDoc, using dense point clouds, and the ground truth (geodetic GNSS receiver) in the east direction for the L’Aquila viaduct.

ΔE—L’Aquila (m)					
Point	101	102	103	104	105
viDoc GL	−0.017	−0.068	−0.064	0.005	−0.032
iPhone dense cloud	0.028	0.001	0.049	0.040	0.058
Xiaomi dense cloud	0.004	−0.039	0.001	0.017	0.033

**Table A2:** Differences between the coordinates acquired by the viDoc ground laser (GL) and mobile phones with viDoc, using dense point clouds, and the ground truth (geodetic GNSS receiver) in the north direction for the L’Aquila viaduct.

ΔN—L’Aquila (m)					
Point	101	102	103	104	105
viDoc GL	−0.044	0.038	0.015	0.029	0.041
iPhone dense cloud	−0.014	−0.007	−0.012	0.009	0.016
Xiaomi dense cloud	−0.008	−0.004	−0.021	−0.031	−0.007

**Table A3:** Differences between the coordinates acquired by the viDoc ground laser (GL) and mobile phones with viDoc, using dense point clouds, and the ground truth (geodetic GNSS receiver) in the height direction for the L’Aquila viaduct.

ΔH—L’Aquila (m)					
Point	101	102	103	104	105
viDoc GL	−0.015	−0.060	0.009	−0.290	−0.134
iPhone dense cloud	0.039	−0.050	0.033	−0.245	−0.008
Xiaomi dense cloud	0.004	−0.084	−0.007	−0.261	−0.039

**Table A4:** Differences between the coordinates acquired by the viDoc ground laser (GL) and mobile phones with viDoc, using dense point clouds, and the ground truth (geodetic GNSS receiver) in the east direction for the Campana viaduct.

ΔE—Campana (m)							
Point	201	202	203	204	205	206	207
viDoc GL	0.026	0.004	−0.026	−0.005	−0.025	0.081	−0.005
iPhone dense cloud	0.007	0.010	0.000	0.013	0.003	−0.013	0.011
Xiaomi dense cloud	−0.009	−0.007	−0.002	−0.012	−0.012	0.003	−0.021

**Table A5:** Differences between the coordinates acquired by the viDoc ground laser (GL) and mobile phones with viDoc, using dense point clouds, and the ground truth (geodetic GNSS receiver) in the north direction for the Campana viaduct.

ΔN—Campana (m)							
Point	201	202	203	204	205	206	207
viDoc GL	−0.026	0.009	0.014	−0.026	−0.050	−0.056	−0.047
iPhone dense cloud	−0.030	−0.020	−0.029	−0.010	−0.026	−0.017	−0.019
Xiaomi dense cloud	−0.015	−0.004	0.002	−0.011	−0.003	0.003	−0.016

**Table A6:** Differences between the coordinates acquired by the viDoc ground laser (GL) and mobile phones with viDoc, using dense point clouds, and the ground truth (geodetic GNSS receiver) in the height direction for the Campana viaduct.

ΔH—Campana (m)							
Point	201	202	203	204	205	206	207
viDoc GL	0.015	−0.019	−0.076	0.003	−0.003	0.012	−0.036
iPhone dense cloud	0.052	0.025	0.024	0.022	0.011	−0.019	−0.011
Xiaomi dense cloud	0.034	0.003	0.022	0.008	0.012	−0.005	0.025

**Table A7:** Differences between the coordinates acquired by the viDoc ground laser (GL) and mobile phones with viDoc, using dense point clouds, and the ground truth (geodetic GNSS receiver) in the east direction for Fontecchio’s Bridge.

ΔE—Fontecchio (m)							
Point	301	302	303	304	305	306	307
viDoc GL	0.011	−0.004	−0.003	−0.036	−0.015	−0.076	0.044
iPhone dense cloud	−0.006	−0.038	−0.030	−0.004	−0.073	−0.015	−0.018
Xiaomi dense cloud	0.252	0.221	−0.043	−0.221	-	0.001	−0.025
**Point**	**308**	**309**	**310**	**311**	**312**	**313**	**314**
viDoc GL	0.020	−0.073	−0.015	0.045	0.008	0.034	0.042
iPhone dense cloud	−0.034	−0.052	−0.019	0.010	−0.008	−0.032	−0.023
Xiaomi dense cloud	0.001	−0.061	−0.081	−0.030	−0.030	−0.022	−0.041

**Table A8:** Differences between the coordinates acquired by the viDoc ground laser (GL) and mobile phones with viDoc, using dense point clouds, and the ground truth (geodetic GNSS receiver) in the north direction for Fontecchio’s Bridge.

ΔN—Fontecchio (m)							
Point	301	302	303	304	305	306	307
viDoc GL	−0.002	0.003	−0.041	−0.169	−0.014	−0.046	−0.009
iPhone dense cloud	−0.010	0.025	−0.031	0.008	−0.016	0.005	0.026
Xiaomi dense cloud	−0.257	−0.164	−0.024	0.032	-	−0.008	−0.049
**Point**	**308**	**309**	**310**	**311**	**312**	**313**	**314**
viDoc GL	−0.033	0.003	0.006	0.012	−0.016	0.043	0.013
iPhone dense cloud	−0.019	0.024	−0.021	−0.038	−0.013	0.000	−0.016
Xiaomi dense cloud	−0.071	−0.050	−0.027	−0.117	−0.098	−0.023	−0.088

**Table A9:** Differences between the coordinates acquired by the viDoc ground laser (GL) and mobile phones with viDoc, using dense point clouds, and the ground truth (geodetic GNSS receiver) in the height direction for Fontecchio’s Bridge.

ΔH—Fontecchio (m)							
Point	301	302	303	304	305	306	307
viDoc GL	0.041	−0.051	−0.006	0.033	−0.007	−0.023	0.101
iPhone dense cloud	0.009	−0.034	−0.072	−0.133	−0.058	−0.022	0.055
Xiaomi dense cloud	−0.141	−0.146	0.008	0.289	-	0.087	0.072
**Point**	**308**	**309**	**310**	**311**	**312**	**313**	**314**
viDoc GL	−0.006	0.020	0.067	0.062	0.042	0.036	−0.003
iPhone dense cloud	−0.086	−0.032	0.061	0.001	−0.013	−0.014	−0.026
Xiaomi dense cloud	−0.012	0.021	0.153	0.094	0.135	0.016	0.034
